# Targeting MicroRNA Function in Acute Pancreatitis

**DOI:** 10.3389/fphys.2017.00726

**Published:** 2017-09-21

**Authors:** Hong Xiang, Xufeng Tao, Shilin Xia, Jialin Qu, Huiyi Song, Jianjun Liu, Dong Shang

**Affiliations:** ^1^College of Integrative Medicine, Dalian Medical University Dalian, China; ^2^Department of General Surgery, First Affiliated Hospital of Dalian Medical University Dalian, China; ^3^College of Pharmacy, Dalian Medical University Dalian, China; ^4^Clinical Laboratory of Integrative Medicine, First Affiliated Hospital of Dalian Medical University Dalian, China

**Keywords:** microRNA, acute pancreatitis, biomarker, target gene, therapeutic tool

## Abstract

Acute pancreatitis (AP) is a common gastrointestinal disorder that featured by acute inflammatory responses leading to systemic inflammatory response syndrome (SIRS) or multiple organ failure. A worldwide increase in annual incidence has been observed during the past decade with high acute hospitalization and mortality. Lack of any specific treatment for AP, even to this day, is a reminder that there is much to be learned about the exact pathogenesis of AP. Fortunately, the discovery of microRNA (miRNA) has started an entirely new thought process regarding the molecular mechanism associated with the disease processes. Given the extensive effort made on miRNA research, certain types of miRNA have been identified across a variety of biological processes, including cell differentiation, apoptosis, metabolism, and inflammatory responses. Mutations in miRNA sequences or deregulation of miRNA expression may contribute to the alteration of a pivotal physiological function leading to AP. Designing miRNA-related tools for AP diagnosis and treatment presents a novel and potential research frontier. In this mini-review, we summarize the current knowledge of various miRNAs closely interacting with AP and the possible development of targeted miRNA therapies in this disease, which may benefit the development of potential disease biomarkers and novel treatment targets for future medical implications.

## Introduction

Acute pancreatitis (AP) is a type of sterile inflammation of the pancreas initiated by dysfunction of the exocrine pancreas that breaks the balance between protective enzymes and stress signals (Lankisch et al., 2015). In the majority of patients, the condition is mild and self-limiting, but ~20–30% of cases eventually develop a severe course with high mortality despite treatment (Bakker et al., 2014). As a rapidly evolving condition, the severity of AP can change quickly within an extremely short amount of time (Mentula and Leppaniemi, 2014). The current management of AP usually consists of combined treatments of nutritional support, analgesics, and protease inhibitors; unfortunately, these therapies exhibit limited efficacy due to their lack of targeting (Tenner et al., 2013; Yokoe et al., 2015). Therefore, it's urgent to search novel diagnostic and therapeutic methods for AP.

The discovery of microRNA (miRNA) has started an entirely new thought process regarding AP diagnosis and treatment. miRNA is a single-stranded non-coding RNA that controls the expression of the majority of genes through either cleavage or translational repression (Iorio and Croce, 2009). There is emerging evidence that altered miRNA expression may lead to the alteration of pivotal physiological functions that participate in inflammation infiltration and complication of multiple diseases, including AP (Hu et al., 2015; Kusnierz-Cabala et al., 2015; Maltby et al., 2016). Therefore, we summarize the interlinked relationships between miRNA and AP to offer a possible diagnostic and therapeutic tool for managing this disease in this mini-review.

## miRNA expression in the pancreas

Many miRNAs are often evolutionarily conserved, and limited in their expression to certain stages in development or to certain cell or tissue types (Sood et al., 2006). Therefore, the ability to determine miRNA expression in the exocrine pancreas will prove valuable in helping to understand the putative roles played by miRNA in AP. Szafranska et al. reported that the expression of miR-216 and miR-217 was identified as a characteristic of human pancreas tissue (Szafranska et al., 2007), which are almost exclusively expressed in rat pancreas (Wang et al., 2017). As normal pancreas consists of ~90% acinar cells, it is easy to think that miR-216 (including highly homologous miR-216a and miR-216b) and miR-217 are enriched in the acinar cells and play a key role in exocrine pancreatic function (Meher et al., 2015; Rouse et al., 2017). Moreover, Kong et al. quantified the relative concentration of miR-216a in pancreas tissues sampled from healthy rats, and found it was 128-fold higher than that in kidney, which had the next highest concentration, indicating that miR-216a may contribute to distinguishing pancreatic diseases from other tissue diseases (Kong et al., 2010). Dixit et al. identified that miR-148a-3p, miR-375-3p, miR-217-5p, and miR-200a-3p are the most abundant miRNAs at basal state, whereas miR-421-3p, miR-24-5p, and miR-29a-5p are the least abundantly expressed in mouse pancreatic acinar cells (Dixit et al., 2016). In addition, Let-7b and miR-495 and their target genes control a transcriptional network that drives pancreatic acinar cell differentiation, which are critical to ensuring acinar homeostasis (Prevot et al., 2013). Due to these correlations of miRNAs in the regulation of physiological processes in pancreas, understanding the modulation of miRNAs expression in AP is crucial.

## Aberrant miRNA expression levels in acute pancreatitis

Early diagnosis of the severity of AP can identify a potential severe acute pancreatitis (SAP) risk as early as possible and provide a high clinical value for improving patient prognosis (Lee et al., 2013). At present, the pancreatic biomarkers amylase and lipase are commonly used in the early prediction of AP, but they are often limited in clinical practice due to their own limitations (Treacy et al., 2001; Huang et al., 2016). Accumulating evidence has shown that abnormal expression of miRNA related to AP pathogenesis can serve as a candidate biomarker of this disease diagnosis and prognosis (Kong et al., 2010; An et al., 2014; Dixit et al., 2016; Zhang et al., 2017). The aberrant miRNA expression patterns in animal models and patients with AP are listed in Table 1.

**Table 1:** Aberrant miRNA expression levels in acute pancreatitis.

**miRNA expressions**	**Samples**	**References**
miR-216a ↑	Rat/patients plasma	Kong et al., 2010; Blenkiron et al., 2014; Zhang et al., 2017
miR-21-3p ↑	Mouse pancreatic acini	Dixit et al., 2016
miR-122 ↑	Mice plasma	Rivkin et al., 2016
miR-216a/miR-216b ↑	Rat Plasma	Endo et al., 2013
miR-216a/miR-375 ↑	Rat serum	Usborne et al., 2014
miR-216a/miR-217 ↑	Rat/mice serum	Goodwin et al., 2014; Wang et al., 2017
miR-216a-5p/miR-375-3p/miR-148a-3p/miR-216b-5p/miR-141-3p ↑	Rat/dog serum	Smith et al., 2016
miR-216a/miR-216b/miR-217 ↑	Rat/canine serum	Calvano et al., 2016; Rouse et al., 2017
miR-375/miR-217/miR-148a/miR-216a/miR-122/miR-214/miR-138 ↑	Rat Mesenteric Lymph	Blenkiron et al., 2014
miR-126-5p/miR-148a-3p/miR-216a-5p/miR-551b-5p/miR-375 ↑	SAP patients serum	Kusnierz-Cabala et al., 2015
miR-216a-5p/miR-551b-5p/miR-375↑	MAP patients serum	Kusnierz-Cabala et al., 2015
miR-92b/miR-10a/miR-7↓	Patients serum	Liu et al., 2014
miR-24-3p/miR-361-5p/ miR-1246/ miR-222-3p↑ miR-181a-5p ↓	HTAP Patients serum	An et al., 2014

### Animals

Based on a comprehensive analysis of miRNA expression profiles, miR-21-3p was significantly overexpressed in mouse pancreatic acinar cells exposed to different pancreatic toxicants (Dixit et al., 2016). Except for tissue specificity, miRNAs can be expressed stably in circulation (Lawrie et al., 2008; Arroyo et al., 2011). It has been reported that the plasma levels of exocrine pancreas-enriched miR-216a, miR-216b, and miR-217 significantly increased after induction of AP or exocrine pancreatic injury (EPIJ) in rodents and dog (Endo et al., 2013; Goodwin et al., 2014; Usborne et al., 2014; Calvano et al., 2016; Rouse et al., 2017; Wang et al., 2017). Smith et al. considered that miR-216a-5p, miR-375-3p, miR-148a-3p, miR-216b-5p, and miR-141-3p continued to elevate in the serum of rats or dogs longer than amylase or lipase and had a large dynamic range (Smith et al., 2016). After cerulein injection, an inverse relationship between plasma levels of miR-122 and erythropoietin (EPO) was observed in mice (Rivkin et al., 2016). Moreover, using an AP rat model, Blenkiron et al. discovered that the expression of miRNAs: miR-375, miR-217, miR-148a, miR-216a, miR-122, miR-214, and miR-138 were increased in mesenteric lymph fluid with a positive correlation with AP severity (Blenkiron et al., 2014). Thus, ever more experimental data regarding AP are currently being generated. However, these discovered numerous miRNA in animals AP are needed further validation and research in clinical settings.

### Patients

In human, serum miR-92b, miR-10a, and miR-7 were reported to reduce during AP, and gave an area under the curve (AUC) value of 0.69 (Liu et al., 2014). Research from Kusnierz-Cabala et al. showed highly abundant miR-126-5p, miR-148a-3p, miR-216a-5p, miR-551b-5p, and miR-375 in the serum of SAP patients, as well as miR-216a-5p, miR-551b-5p, and miR-375 in mild acute pancreatitis (MAP) patients. Further, receiver operating characteristic (ROC) curve analysis revealed that miR-126-p and miR-551b-5p may be used as potential markers for predicting AP severity with a good AUC (sensitivity = 60.0%, specificity = 87.1%; and sensitivity = 69.2%, specificity = 72.6%; Kusnierz-Cabala et al., 2015). During hypertriglyceridemia- induced AP (HTAP), An et al. observed significant up-regulation in the serum concentration of the following miRNAs: miR24-3p, miR-361-5p, miR-1246, and miR-222-3p; miR-181a-5p were constantly down-regulated. The AUC for all five overlapping miRNA were 0.889 (sensitivity = 100%; specificity = 83.3%), 0.722 (sensitivity = 85%; specificity = 90%), 0.917 (sensitivity = 98%; specificity = 92%), 0.833 (sensitivity = 100%; specificity = 83.3%), and 0.972 (sensitivity = 100%; specificity = 83.3%), successively. These results demonstrated that the expressions of these miRNAs, especially miR-181a-5p, can be used to accurately evaluate the progression of HTAP. Moreover, miR-181a-5p has a positive correlation with Ca^2+^ but a negative correlation with triglyceride (TG), total cholesterol (TC), and fasting blood glucose (FBG) (An et al., 2014). In summary, as shown in Table 1, these clinical studies indicate that the potential miRNA biomarkers of AP include miR-92b, miR-126-5p, miR24-3p, miR-181a-5p, and so on.

Due to the specificity and sensitivity of miRNA in the regulation of the AP process, it's better than conventional biomarkers with poor tissue and cell specificity (Beuvink et al., 2007; Tombol et al., 2009). Although, it is generally accepted that RNA is easily degraded when it is isolated from RNase-rich tissue samples, such as the pancreas, Kim et al. believed that RNA degradation due to extended storage at room temperature has no effect on the predictive power of the tissue-specific miRNA quantitative reverse transcription polymerase chain reaction (QRTPCR) predictor (Kim et al., 2011). Furthermore, several miRNAs are able to withstand a variety of harsh environments, such as repeated freezing and thawing or high acidic or basic environments with extreme pH (Machado et al., 2015). Compared with other biomarkers, miRNA expression is stable in body fluids, especially in the bloodstream, generating interest in the utilization of miRNA as serum- and/or plasma-based biomarkers of AP diagnosis and patient stratification (Mitchell et al., 2008; Kong et al., 2010; Liu et al., 2014). However, various miRNAs are abnormally presented in the disease condition of AP; thus, a detailed miRNA profile should be created that also refers to other diagnostic methods to avoid misinterpretation.

## miRNA regulating acute pancreatitris-related gene expression

**Figure 1:**
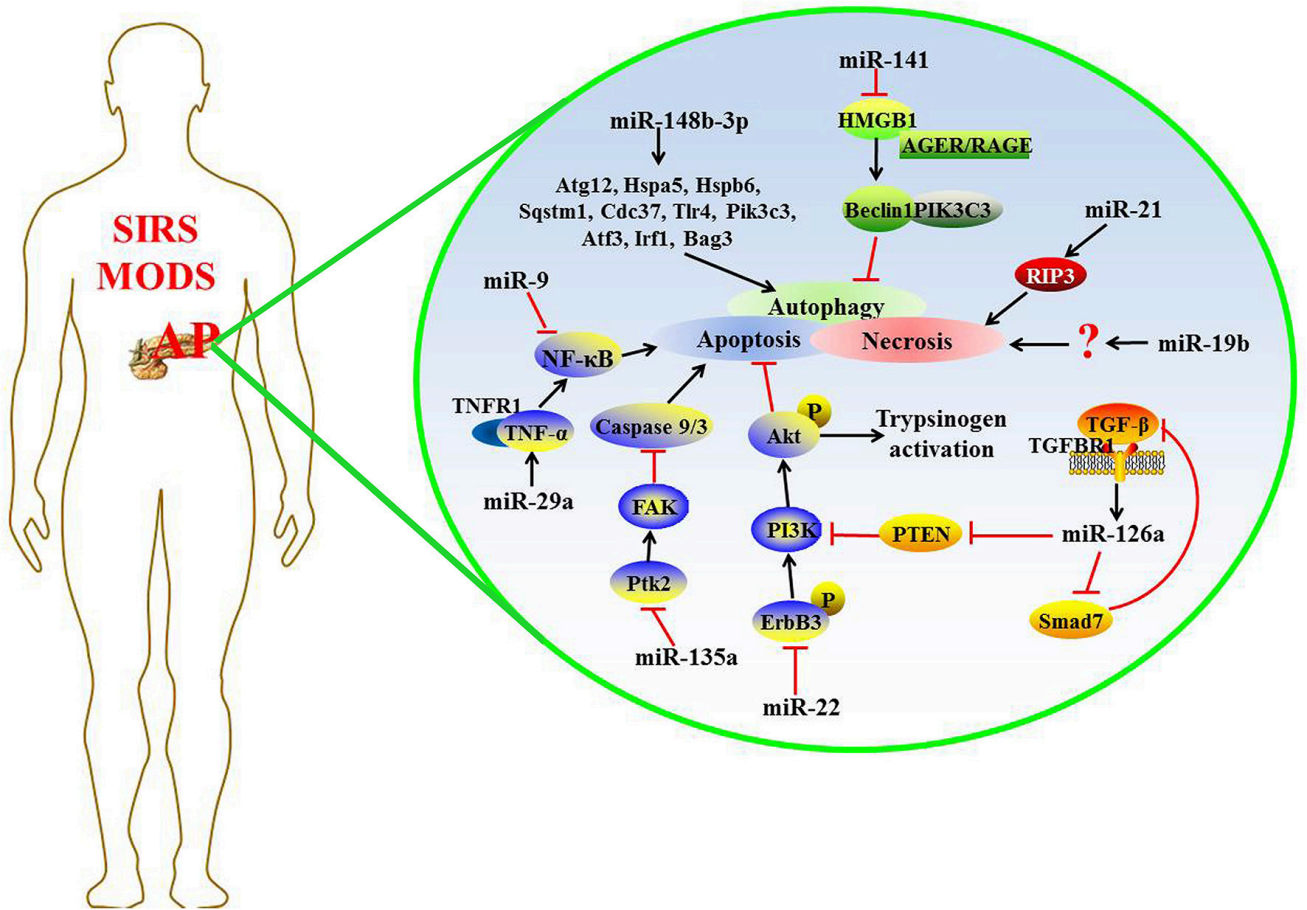
The possible mechanisms of miRNAs regulating target expression in complicated AP.

Recently, some miRNAs were found to regulate the expression of target genes in complicated AP, and the detailed mechanism is shown in Figure 1.

### Apoptosis/necrosis

Apoptosis and necrosis are the two main patterns of pancreatic acinar cell death during AP, and they may interchange with one another under appropriate conditions (Bhatia, 2004; Mareninova et al., 2006). Apoptosis maintains membrane integrity without stimulating the immune system, whereas necrotic cells release damage- associated molecular pattern molecules (DAMPs) that trigger an inflammatory cascade (Bhatia, 2004).

Data from *in vivo* and *in vitro* models of acute edematous pancreatitis (AEP) have shown that the expression levels of miR-22 and miR-135a increased significantly compared with the normal group, which play a protective role in AEP through the pro-apoptosis activity in pancreatic acinar cells by inhibiting the expression of their target gene, ErbB3 and Ptk2, respectively (Qin et al., 2014). Tyrosine-phosphorylated ErbB3 shows higher affinity for phosphatidylinositol-4, 5-bisphosphate 3-kinase (PI3K), so activated ErbB3 leads to a strong activation of the PI3K/serine/threonine kinase (Akt) signaling pathway that can result in apoptosis resistance (Soltoff et al., 1994; Roy et al., 2010). The activation of PI3K/Akt is also necessary for activation of intrapancreatic trypsinogen (Fischer et al., 2007). Ptk2 gene encodes focal adhesion kinase (FAK) protein, and the activation of this gene may be an early important step in cell death (Alisi et al., 2012). Many studies have demonstrated that genetic ablation of Ptk2 and reducing the FAK protein level could promote drug-inducing apoptosis via markedly enhancing caspase 9 and caspase 3 activities (Yuan et al., 2010; Xie et al., 2011; Shi et al., 2012). Evidence from the study of Fu et al. had demonstrated that apoptotic rate and TNFRSF1A expression were obviously elevated following the up-regulation of miR-29a expression in AR42J cells treated with recombinant rat TNF-α (Fu et al., 2016). TNFRSF1A, as the target gene of miR-29a, encodes the tumor necrosis factor receptor 1 (TNFR1) protein that is the major signaling receptor for TNF-α (Borghini et al., 2011). The combination of TNF-α to TNFR1 immediately activates nuclear factor-kappa B (NF-κB) and subsequent apoptosis (Wajant et al., 2003).

The necrosis rate of pancreatic acinar cells and the following inflammation correlate with the mortality of patients with acute necrotizing pancreatitis (ANP) (Xu et al., 2015). Increasing research has demonstrated the function of miRNAs in pancreatic acinar cell necrosis, indicating that miRNA may be a potential target for drug development against AP (Ma et al., 2015; Hu et al., 2016). The increased expression of miR-19b in ANP rats or taurolithocholic acid 3-sulfate disodium salt (TLC-S)-treated AR42J cells, could promote cellular necrosis; otherwise, deleting miR-19b could decrease the necrosis rate (Hu et al., 2016). Ma et al. report that miR-21 is overexpressed in a mouse model of AP; miR-21 inhibition protects against caerulein- or L-arginine-induced AP and effectively reduced disease severity. Further, silencing the expression of miR-21 is protective in TNF-induced SIRS. MiR-21 promotes TNF-α-induced necroptosis, a pathologic condition involving receptor-interacting protein 3 (RIP3)-dependent regulated necrosis (Ma et al., 2015). RIP3 is a component of the necrosome, which can be directly cleaved and inactivated by caspase 8 to negatively regulate necroptosis induced by TNF-α (Kaiser et al., 2011). Thus, miRNAs critically involved in the apoptosis and/or necrosis processes of AP.

### Autophagy

Autophagy is a form of programmed cell death in the evolutionary process, and the main function of it is lysosomal self-digestion of a cell's own components in response to external stimuli (Jones et al., 2013; Vernon and Tang, 2013). Many studies have shown that autophagy is involved in the progression of AP, but its function in the course of AP is still controversial. Previous studies reported that selective autophagy plays a cytoprotective role by eliminating excess zymogen activation, and reduces pancreatic cell death induced by trypsin at the onset of AP (Grasso et al., 2011). Another opposite view is that autophagy contributes to the formation of acinar cell vacuoles and to the activation of trypsinogen as the primary mechanism underlying the pathogenesis of AP (Gukovsky et al., 2012).

miRNA preserves the autophagy process via regulating the expression of autophagy-associated genes. Using an *in vitro* acinar cell autophagy model, Gao et al. identified that downregulated miR-148b-3p may be a moderator in the autophagic process, and 10 autophagy-related genes among miR-148b-3p targets were identified through data mining technology: Sqstm1, Cdc37, Tlr4, Atg12, Hspa5, Hspb6, Pik3c3, Atf3, Irf1, and Bag3. These target genes indirectly affected the regulation of autophagy by interfering with key genes related to the autophagic signaling pathway (Gao et al., 2016). However, further exploration is required to confirm the regulatory effects and mechanism of miR-148b-3p and its target genes on the autophagic process. Recent research reported that in L-arginine-induced AP mice, the pancreatic tissue injury in mice using miR-141 adenoviral vector treatment is much less than normal saline treatment, as are autophagosomal and autolysosomal processes in pancreatic tissues. Mechanistically, miR-141 antagonizes high mobility group box-1 protein (HMGB1) expression and further reduces the downstream protein Beclin-1 level, leading to a block in the process of autophagosomes formation, suggesting that miR-141 may inhibit autophagy through the HMGB1/Beclin-1 pathway (Zhu et al., 2016). HMGB1 is a conserved nuclear protein that plays subcellular localization-dependent roles in the regulation of autophagy (Tang et al., 2012). Cytosolic HMGB1 is a novel Beclin-1-binding protein to induce autophagy (Tang et al., 2010). Extracellular HMGB1 combines with advanced glycosylation end product-specific receptor (AGER/RAGE) to accelerate the formation of Beclin-1- phosphatidylinositol 3-kinase catalytic subunit type 3 (PIK3C3) core autophagic complexes (Kang et al., 2010; Yu et al., 2016). These findings show that miRNA appears to be a promising candidate for study on the mechanisms of autophagy-promoted AP and the gene therapy of AP (Zhu et al., 2016).

### TGF-β feedback pathway

Transforming growth factor-β (TGF-β) is a multifunctional cytokine that participates in a variety of biological processes, including cell apoptosis and immune system regulation (Mikami et al., 2006). It has been reported that TGF-β and its receptors are up-regulated in patients with AP (Friess et al., 1998; Wildi et al., 2007). The expression of miR-216a increases in a dose-dependent manner in AR42J cells after TGF-β stimulation. Meanwhile, the TGF-β inhibitor SB431542 can decrease the miR-216a expression in pancreatic tissue and serum in the cerulean-induced AP mouse model. TGF-β aggravates AP via up-regulating miR-216a, which targets PTEN and Smad7 (Zhang et al., 2015). PTEN is a suppressor of the PI3K/Akt signaling pathway associated with cell apoptosis (Blanco-Aparicio et al., 2007). Smad7 blocks the TGF-β signaling pathway through a negative feedback loop, and it also acts as a cross talk mediator between TGF-β signaling and others (Yan and Chen, 2011). Therefore, TGF-β/miR-216a signaling pathway regulates multiple biological processes in AP.

### NF-κB signaling pathway

Mesenchymal stem cells (MSCs) belong to the stem cell family and have been widely used as gene delivery vehicle of delivering exogenous genes to damaged tissues for a cell-base therapeutic strategy (Si et al., 2011). MiR-9 is a key paracrine molecular of bone marrow-derived mesenchymal stem cells (BMSCs), and the up-regulation of it could inhibit the inflammatory response induced by lipopolysaccharide (LPS) in human polymorphonuclear neutrophils (PMN) and peripheral blood mononuclear cells (PBMC) (Bazzoni et al., 2009). Qian et al. found that miR-9-modified BMSCs (pri-miR-9-BMSCs) significantly reduced pancreatic injury and the activities of serum amylase and lipase in AP rats. Meanwhile, decreased release of inflammatory factors and enhanced regeneration of the necrotized pancreatic tissues are also observed. These results show that miR-9 may be an anti-inflammatory factor involved in the progression of AP. BMSCs deliver miR-9 to the damaged pancreas or PBMCs, which may attenuate SAP targeting of the NF-κB1/p50 gene (Qian et al., 2017).

When AP occurs, the damaged pancreatic cells release pro-inflammatory mediators to stimulate macrophages in the pancreas, peritoneum, and other tissues (Gutierrez et al., 2008). Activated macrophages secrete various inflammatory cytokines, resulting in the spread of inflammation (Jaffray et al., 2000; Ni et al., 2014). Therefore, cell-level therapies targeting macrophages may achieve a valuable breakthrough in the treatment of AP. It has been discovered that intercellular communication plays an essential role in the activation of pancreatitis-associated macrophages (Lundberg et al., 2000). miRNA is thought to be a mediator of intercellular communication that is transported to recipient cells to regulate their functions (Chevillet et al., 2014). Recently, the results from Zhao et al.'s research demonstrated that activated AR42J cells enhanced NF-κB activation in the macrophages by secreting exosomes carrying differentially expressed miRNAs (Zhao et al., 2016). These researches indicate that miRNA appears to regulate the NF-κB promoted-inflammatory responses in AP.

## miRNA as a potential therapeutic tool

miRNA play key roles in gene expression regulation; thus, manipulating miRNA function *in vitro* and *in vivo* is a potential therapy at the genetic level to modulate disease pathogenesis. The discovery of miRNA inhibitors (e.g., anti-miRNAs and miRNA sponges) or enhancers (mimics) interfere with miRNA, increasing or inhibiting translation of miRNA-targeted mRNAs, thus modifying protein expression levels and greatly pushing forward the development of novel drugs (Ebert et al., 2007; Lennox and Behlke, 2011; Robb et al., 2017). Currently, a major restriction to exploiting miRNAs as therapies is the likelihood of undesired side effects due to their biological properties, in which individual miRNAs modulate multiple downstream targets, and interfering with single miRNAs can have broad impacts on multiple cellular pathways simultaneously and will potentially offset the desired therapeutic effects, particularly when systemic drug delivery is used (Baker, 2010). Therefore, a personalized miRNA therapy to maintain other functions without disturbance and optimize drug delivery systems are required in future research.

## Conclusion

In this mini-review, we have discussed the promising role of miRNAs in developing more effective therapies for AP apart from acting as potential diagnostic tools. Although, some miRNAs have been discovered to demonstrate a direct or indirect link to AP progression, investigations on applying miRNA in AP treatment remain at the development stage. These basic researches can provide a large number of valuable information for the clinical promotion of miRNA-diagnostic application and the future development of AP-treatment.

## Author contributions

HX, XT, SX, JQ, HS, JL, and DS wrote the manuscript.

### Conflict of interest statement

The authors declare that the research was conducted in the absence of any commercial or financial relationships that could be construed as a potential conflict of interest.
